# *N*-[(*E*)-Anthracen-9-yl­methyl­idene]-3,4-dimethyl-1,2-oxazol-5-amine

**DOI:** 10.1107/S1600536811050471

**Published:** 2011-11-30

**Authors:** Abdullah M. Asiri, Abdulrahman O. Al-Youbi, Salman A. Khan, M. Nawaz Tahir

**Affiliations:** a Department of Chemistry, Faculty of Science, King Abdulaziz University, Jeddah 21589, PO Box 80203, Saudi Arabia; b The Center of Excellence for Advanced Materials Reesrch, King Abdulaziz University, Jeddah 21589, PO Box 80203, Saudi Arabia; c University of Sargodha, Department of Physics, Sargodha, Pakistan

## Abstract

In the title compound, C_20_H_16_N_2_O, an intra­molecular C—H⋯N forms an *S*(6) ring motif. In the crystal, the mol­ecules are stacked with their anthracene ring planes in sheets along [100].

## Related literature

For applications of compounds containing azomethine groups, see: Khuhawar *et al.* (2004 ▶). Schiff base compounds demonstrate anti­bacterial (Asiri & Khan, 2010 ▶), anti­tumor activity (Saxena & Tandon, 1983 ▶) and anti-HIV activity (Pandeya *et al.*, 1999 ▶). For related structures, see: Asiri *et al.* (2011**a* ▶,b* ▶). For graph-set notation, see: Bernstein *et al.* (1995 ▶).
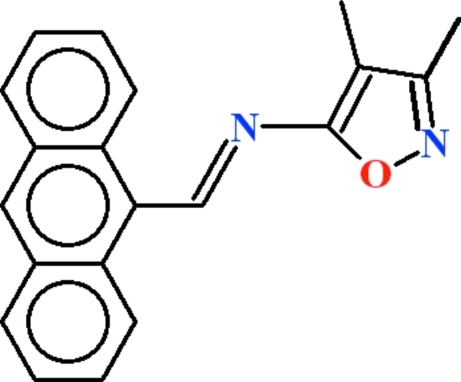


## Experimental

### 

#### Crystal data

C_20_H_16_N_2_O
*M* *_r_* = 300.35
Monoclinic, 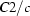

*a* = 22.4919 (14) Å
*b* = 6.1666 (4) Å
*c* = 22.6801 (13) Å
β = 102.015 (2)°
*V* = 3076.8 (3) Å^3^
*Z* = 8
Mo *K*α radiation
μ = 0.08 mm^−1^
*T* = 296 K
0.32 × 0.24 × 0.22 mm

#### Data collection

Bruker KAPPA APEXII CCD diffractometer
Absorption correction: multi-scan (*SADABS*; Sheldrick, 2004 ▶) *T*_min_ = 0.975, *T*_max_ = 0.980
12925 measured reflections
3193 independent reflections
2381 reflections with *I* > 2σ(*I*)
*R*_int_ = 0.028

#### Refinement

*R*[*F* ^2^ > 2σ(*F* ^2^)] = 0.044
*wR*(*F* ^2^) = 0.130
*S* = 1.04
3193 reflections
210 parameters
H-atom parameters constrained
Δρ_max_ = 0.26 e Å^−3^
Δρ_min_ = −0.21 e Å^−3^

### 

Data collection: *APEX2* (Bruker, 2009 ▶); cell refinement: *SAINT* (Bruker, 2009 ▶); data reduction: *SAINT*; program(s) used to solve structure: *SHELXS97* (Sheldrick, 2008 ▶); program(s) used to refine structure: *SHELXL97* (Sheldrick, 2008 ▶); molecular graphics: *ORTEP-3 for Windows* (Farrugia, 1997 ▶) and *PLATON* (Spek, 2009 ▶); software used to prepare material for publication: *WinGX* (Farrugia, 1999 ▶) and *PLATON*.

## Supplementary Material

Crystal structure: contains datablock(s) global, I. DOI: 10.1107/S1600536811050471/fk2045sup1.cif

Structure factors: contains datablock(s) I. DOI: 10.1107/S1600536811050471/fk2045Isup2.hkl

Supplementary material file. DOI: 10.1107/S1600536811050471/fk2045Isup3.cml

Additional supplementary materials:  crystallographic information; 3D view; checkCIF report

## Figures and Tables

**Table 1:** Hydrogen-bond geometry (Å, °)

*D*—H⋯*A*	*D*—H	H⋯*A*	*D*⋯*A*	*D*—H⋯*A*
C2—H2⋯N1	0.93	2.20	2.840 (2)	125

**Fig. 1.**
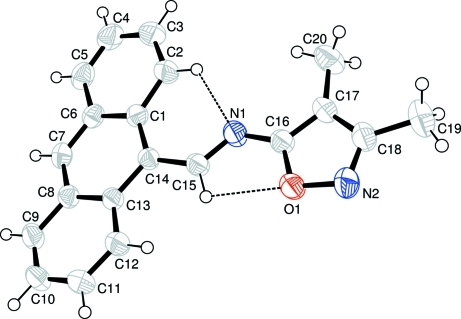
View of the title compound with the atom numbering scheme. The thermal ellipsoids are drawn at the 50% probability level. H-atoms are shown as small spheres of arbitrary radii. The dotted lines represent the intra-molecular H-bonds.

## supplementary crystallographic information

### Comment

Compounds containing azomethine groups (C═N) play a vital role in chemistry (Khuhawar *et al.*, 2004). Schiff-base compounds have been used as fine chemicals and medical substrates such as intermediates for the various reactions and antibacterial (Asiri & Khan, 2010), antitumor activity (Saxena & Tandon, 1983) and anti-HIV activity (Pandeya *et al.*, 1999). Schiff bases containing heterocyclic rings dramatically increase the biological activity. The crystal structure of title compound (I), (Fig. 1) is being reported here.

Recently, we have reported the crystal structure of (II) *i.e.*, 4-[(anthracen-9-ylmethylidene)amino]-1,5-dimethyl-2-phenyl-1*H*- pyrazol-3(2*H*)-one (Asiri *et al.*, 2011*a*) and (III) *i.e.*, *N*-[(*E*)-1,3-benzodioxol-5-ylmethylidene]-3,4-dimethyl-1,2-oxazol -5-amine (Asiri *et al.*, 2011*b*) which contain the common moieties of (I).

In (I), the anthracen rings A (C1–C6), B (C1/C6/C7/C8/C13/C14) and C (C8–C13) are planar with r. m. s. deviations of 0.0090, 0.0241 and 0.0063 Å, respectively. The dihedral angles A/B, A/C and B/C are 4.80 (11)°, 8.36 (11) ° and 3.90 (10) °, respectively. The 3,4-dimethyl-1,2 -oxazol-5-amine moiety D (N1/C16–C20/N2/O1) is also planar with r. m. s. deviation of 0.0061 Å. The dihedral angles A/D, B/D and C/D are 7.59 (10)°, 3.85 (10)° and 5.48 (10)°, respectively. Intra-molecular H-bonds of C—H···N and C—H···O type complete S(6) and S(5) ring motifs (Fig. 1)(Bernstein *et al.*, 1995). The crystal packing shows the anthracen ring planes stacked in parallel sheets along [100].

### Experimental

A mixture of anthracene-9-carbaldehyde (0.50 g, 2.4 mmol) and 5-amino-3,4-dimethylisoxazole (2.4 mmol) in ethanol (15 ml) was heated for 3 h. The progress of the reaction was monitored by TLC. The solid that separated from the cooled mixture was collected and recrystallized from a methanol:chloroform mixture (8:2) to give red prisms of (I).

Red solid: Yield: 82%, m.p. 419–420 K.

### Refinement

Aromatic H-atoms were positioned geometrically (C–H = 0.93Å) and refined as riding with *U*_iso_(H) = *1.2U*_eq_(C); methyl H positions were derived from difference maps (HFIX 137) and refined with C–H = 0.96Å and *U*_iso_(H) = *1.5U*_eq_(C)

### Crystal data

**Table:**

C_20_H_16_N_2_O	*F*(000) = 1264
*M**_r_* = 300.35	*D*_x_ = 1.297 Mg m^−^^3^
Monoclinic, *C*2/*c*	Mo *K*α radiation, λ = 0.71073 Å
Hall symbol: -C 2yc	Cell parameters from 2381 reflections
*a* = 22.4919 (14) Å	θ = 1.9–26.5°
*b* = 6.1666 (4) Å	µ = 0.08 mm^−^^1^
*c* = 22.6801 (13) Å	*T* = 296 K
β = 102.015 (2)°	Prism, red
*V* = 3076.8 (3) Å^3^	0.32 × 0.24 × 0.22 mm
*Z* = 8	

### Data collection

**Table:**

Bruker KAPPA APEXII CCD diffractometer	3193 independent reflections
Radiation source: fine-focus sealed tube	2381 reflections with *I* > 2σ(*I*)
graphite	*R*_int_ = 0.028
Detector resolution: 8.10 pixels mm^-1^	θ_max_ = 26.5°, θ_min_ = 1.9°
ω scans	*h* = −28→27
Absorption correction: multi-scan (*SADABS*; Sheldrick, 2004)	*k* = −7→7
*T*_min_ = 0.975, *T*_max_ = 0.980	*l* = −23→28
12925 measured reflections	

### Refinement

**Table:**

Refinement on *F*^2^	Primary atom site location: structure-invariant direct methods
Least-squares matrix: full	Secondary atom site location: difference Fourier map
*R*[*F*^2^ > 2σ(*F*^2^)] = 0.044	Hydrogen site location: geom and difmap
*wR*(*F*^2^) = 0.130	H-atom parameters constrained
*S* = 1.04	*w* = 1/[σ^2^(*F*_o_^2^) + (0.0637*P*)^2^ + 1.2672*P*] where *P* = (*F*_o_^2^ + 2*F*_c_^2^)/3
3193 reflections	(Δ/σ)_max_ < 0.001
210 parameters	Δρ_max_ = 0.26 e Å^−^^3^
0 restraints	Δρ_min_ = −0.21 e Å^−^^3^

### Special details

**Table:**

Geometry. Bond distances, angles *etc*. have been calculated using the rounded fractional coordinates. All su's are estimated from the variances of the (full) variance-covariance matrix. The cell e.s.d.'s are taken into account in the estimation of distances, angles and torsion angles
Refinement. Refinement of *F*^2^ against ALL reflections. The weighted *R*-factor *wR* and goodness of fit *S* are based on *F*^2^, conventional *R*-factors *R* are based on *F*, with *F* set to zero for negative *F*^2^. The threshold expression of *F*^2^ > σ(*F*^2^) is used only for calculating *R*-factors(gt) *etc*. and is not relevant to the choice of reflections for refinement. *R*-factors based on *F*^2^ are statistically about twice as large as those based on *F*, and *R*- factors based on ALL data will be even larger.

### Fractional atomic coordinates and isotropic or equivalent isotropic displacement parameters (Å^2^)

**Table:**

	*x*	*y*	*z*	*U*_iso_*/*U*_eq_	
O1	0.09676 (5)	0.50025 (19)	0.05196 (5)	0.0516 (4)	
N1	0.10276 (6)	0.2014 (2)	0.11940 (6)	0.0459 (4)	
N2	0.05953 (7)	0.6861 (3)	0.03694 (7)	0.0584 (5)	
C1	0.16650 (6)	−0.1894 (3)	0.17670 (6)	0.0379 (4)	
C2	0.12182 (7)	−0.1253 (3)	0.20964 (7)	0.0486 (5)	
C3	0.10875 (8)	−0.2518 (3)	0.25450 (8)	0.0580 (6)	
C4	0.13754 (8)	−0.4522 (3)	0.26932 (8)	0.0606 (7)	
C5	0.17975 (8)	−0.5201 (3)	0.23969 (8)	0.0528 (6)	
C6	0.19672 (7)	−0.3919 (3)	0.19346 (7)	0.0403 (5)	
C7	0.24311 (7)	−0.4589 (3)	0.16590 (7)	0.0429 (5)	
C8	0.26386 (7)	−0.3322 (3)	0.12393 (6)	0.0393 (5)	
C9	0.31446 (7)	−0.3980 (3)	0.09933 (7)	0.0497 (6)	
C10	0.33622 (8)	−0.2712 (3)	0.06009 (8)	0.0556 (6)	
C11	0.30869 (8)	−0.0702 (3)	0.04332 (8)	0.0554 (6)	
C12	0.25992 (7)	−0.0016 (3)	0.06472 (7)	0.0476 (5)	
C13	0.23446 (6)	−0.1286 (2)	0.10601 (6)	0.0365 (5)	
C14	0.18386 (6)	−0.0631 (2)	0.13028 (6)	0.0356 (4)	
C15	0.15128 (7)	0.1314 (2)	0.10573 (7)	0.0398 (5)	
C16	0.07545 (7)	0.3889 (3)	0.09501 (7)	0.0415 (5)	
C17	0.02670 (7)	0.4911 (3)	0.10808 (7)	0.0447 (5)	
C18	0.01918 (7)	0.6751 (3)	0.07070 (8)	0.0501 (6)	
C19	−0.02801 (9)	0.8473 (3)	0.06716 (10)	0.0733 (8)	
C20	−0.00958 (9)	0.4243 (4)	0.15280 (9)	0.0675 (7)	
H2	0.10126	0.00518	0.20030	0.0584*	
H3	0.08009	−0.20426	0.27582	0.0696*	
H4	0.12735	−0.53754	0.29956	0.0728*	
H5	0.19841	−0.65363	0.24946	0.0633*	
H7	0.26093	−0.59368	0.17593	0.0515*	
H9	0.33285	−0.53105	0.11056	0.0596*	
H10	0.36913	−0.31682	0.04434	0.0667*	
H11	0.32424	0.01809	0.01692	0.0666*	
H12	0.24256	0.13187	0.05217	0.0571*	
H15	0.16724	0.21079	0.07779	0.0478*	
H19A	−0.02371	0.91685	0.10569	0.1099*	
H19B	−0.06768	0.78325	0.05612	0.1099*	
H19C	−0.02301	0.95261	0.03740	0.1099*	
H20A	0.00725	0.29388	0.17266	0.1013*	
H20B	−0.05088	0.39842	0.13254	0.1013*	
H20C	−0.00855	0.53733	0.18209	0.1013*	

### Atomic displacement parameters (Å^2^)

**Table:**

	*U*^11^	*U*^22^	*U*^33^	*U*^12^	*U*^13^	*U*^23^
O1	0.0536 (7)	0.0504 (7)	0.0565 (7)	0.0142 (5)	0.0243 (5)	0.0109 (5)
N1	0.0428 (7)	0.0438 (8)	0.0535 (8)	0.0056 (6)	0.0158 (6)	0.0045 (6)
N2	0.0621 (9)	0.0510 (9)	0.0640 (10)	0.0189 (7)	0.0177 (8)	0.0137 (7)
C1	0.0335 (7)	0.0412 (8)	0.0383 (8)	−0.0033 (6)	0.0061 (6)	−0.0001 (6)
C2	0.0451 (9)	0.0550 (10)	0.0495 (9)	0.0033 (8)	0.0184 (7)	0.0062 (8)
C3	0.0509 (10)	0.0745 (13)	0.0539 (10)	−0.0015 (9)	0.0232 (8)	0.0084 (9)
C4	0.0559 (11)	0.0746 (13)	0.0533 (10)	−0.0048 (10)	0.0157 (8)	0.0243 (10)
C5	0.0492 (10)	0.0527 (10)	0.0539 (10)	−0.0015 (8)	0.0051 (8)	0.0167 (8)
C6	0.0380 (8)	0.0401 (9)	0.0403 (8)	−0.0045 (7)	0.0022 (6)	0.0036 (7)
C7	0.0411 (8)	0.0374 (8)	0.0472 (9)	0.0045 (7)	0.0021 (7)	0.0024 (7)
C8	0.0360 (8)	0.0410 (9)	0.0394 (8)	0.0027 (6)	0.0043 (6)	−0.0051 (7)
C9	0.0426 (9)	0.0536 (10)	0.0522 (10)	0.0125 (8)	0.0085 (7)	−0.0051 (8)
C10	0.0421 (9)	0.0734 (13)	0.0548 (10)	0.0113 (9)	0.0180 (8)	−0.0067 (9)
C11	0.0489 (10)	0.0699 (12)	0.0530 (10)	0.0021 (9)	0.0232 (8)	0.0059 (9)
C12	0.0455 (9)	0.0513 (10)	0.0489 (9)	0.0067 (8)	0.0168 (7)	0.0078 (8)
C13	0.0341 (8)	0.0395 (9)	0.0356 (7)	0.0008 (6)	0.0064 (6)	−0.0028 (6)
C14	0.0329 (7)	0.0372 (8)	0.0368 (7)	0.0000 (6)	0.0076 (6)	−0.0010 (6)
C15	0.0392 (8)	0.0405 (9)	0.0429 (8)	0.0026 (7)	0.0157 (6)	0.0023 (7)
C16	0.0407 (8)	0.0419 (9)	0.0437 (8)	0.0021 (7)	0.0132 (7)	0.0006 (7)
C17	0.0396 (8)	0.0453 (9)	0.0507 (9)	0.0041 (7)	0.0127 (7)	−0.0054 (7)
C18	0.0442 (9)	0.0495 (10)	0.0553 (10)	0.0090 (8)	0.0077 (8)	−0.0045 (8)
C19	0.0640 (13)	0.0621 (13)	0.0937 (16)	0.0253 (10)	0.0162 (11)	−0.0007 (11)
C20	0.0554 (11)	0.0771 (14)	0.0791 (13)	0.0055 (10)	0.0347 (10)	−0.0032 (11)

### Geometric parameters (Å, °)

**Table:**

O1—N2	1.418 (2)	C14—C15	1.4545 (19)
O1—C16	1.360 (2)	C16—C17	1.350 (2)
N1—C15	1.271 (2)	C17—C18	1.405 (3)
N1—C16	1.371 (2)	C17—C20	1.486 (3)
N2—C18	1.305 (2)	C18—C19	1.492 (3)
C1—C2	1.427 (2)	C2—H2	0.9300
C1—C6	1.435 (3)	C3—H3	0.9300
C1—C14	1.428 (2)	C4—H4	0.9300
C2—C3	1.362 (2)	C5—H5	0.9300
C3—C4	1.403 (3)	C7—H7	0.9300
C4—C5	1.339 (3)	C9—H9	0.9300
C5—C6	1.427 (2)	C10—H10	0.9300
C6—C7	1.386 (2)	C11—H11	0.9300
C7—C8	1.385 (2)	C12—H12	0.9300
C8—C9	1.427 (2)	C15—H15	0.9300
C8—C13	1.438 (2)	C19—H19A	0.9600
C9—C10	1.351 (2)	C19—H19B	0.9600
C10—C11	1.402 (3)	C19—H19C	0.9600
C11—C12	1.356 (2)	C20—H20A	0.9600
C12—C13	1.429 (2)	C20—H20B	0.9600
C13—C14	1.4220 (19)	C20—H20C	0.9600
			
N2—O1—C16	107.56 (12)	N2—C18—C19	120.35 (17)
C15—N1—C16	121.58 (14)	C17—C18—C19	127.02 (16)
O1—N2—C18	105.36 (15)	C1—C2—H2	119.00
C2—C1—C6	116.67 (14)	C3—C2—H2	119.00
C2—C1—C14	124.43 (15)	C2—C3—H3	119.00
C6—C1—C14	118.88 (13)	C4—C3—H3	119.00
C1—C2—C3	121.16 (16)	C3—C4—H4	120.00
C2—C3—C4	121.52 (17)	C5—C4—H4	120.00
C3—C4—C5	119.68 (17)	C4—C5—H5	119.00
C4—C5—C6	121.43 (17)	C6—C5—H5	119.00
C1—C6—C5	119.50 (15)	C6—C7—H7	119.00
C1—C6—C7	119.97 (15)	C8—C7—H7	119.00
C5—C6—C7	120.51 (16)	C8—C9—H9	119.00
C6—C7—C8	122.32 (17)	C10—C9—H9	119.00
C7—C8—C9	121.24 (16)	C9—C10—H10	120.00
C7—C8—C13	119.07 (14)	C11—C10—H10	120.00
C9—C8—C13	119.68 (14)	C10—C11—H11	119.00
C8—C9—C10	121.40 (17)	C12—C11—H11	119.00
C9—C10—C11	119.36 (17)	C11—C12—H12	119.00
C10—C11—C12	121.53 (17)	C13—C12—H12	119.00
C11—C12—C13	121.87 (16)	N1—C15—H15	117.00
C8—C13—C12	116.13 (13)	C14—C15—H15	117.00
C8—C13—C14	119.78 (12)	C18—C19—H19A	109.00
C12—C13—C14	124.07 (12)	C18—C19—H19B	109.00
C1—C14—C13	119.62 (12)	C18—C19—H19C	109.00
C1—C14—C15	122.60 (13)	H19A—C19—H19B	109.00
C13—C14—C15	117.78 (12)	H19A—C19—H19C	109.00
N1—C15—C14	125.21 (14)	H19B—C19—H19C	109.00
O1—C16—N1	121.36 (14)	C17—C20—H20A	109.00
O1—C16—C17	110.22 (15)	C17—C20—H20B	109.00
N1—C16—C17	128.42 (15)	C17—C20—H20C	109.00
C16—C17—C18	104.22 (14)	H20A—C20—H20B	109.00
C16—C17—C20	127.36 (17)	H20A—C20—H20C	109.00
C18—C17—C20	128.41 (17)	H20B—C20—H20C	109.00
N2—C18—C17	112.63 (16)		
			
C16—O1—N2—C18	−0.22 (18)	C6—C7—C8—C13	−3.1 (2)
N2—O1—C16—N1	−179.18 (14)	C7—C8—C9—C10	−177.53 (16)
N2—O1—C16—C17	0.46 (18)	C13—C8—C9—C10	1.1 (2)
C16—N1—C15—C14	178.72 (14)	C7—C8—C13—C12	177.20 (14)
C15—N1—C16—O1	4.6 (2)	C7—C8—C13—C14	−1.6 (2)
C15—N1—C16—C17	−174.96 (17)	C9—C8—C13—C12	−1.5 (2)
O1—N2—C18—C17	−0.09 (19)	C9—C8—C13—C14	179.74 (14)
O1—N2—C18—C19	179.65 (15)	C8—C9—C10—C11	0.3 (3)
C6—C1—C2—C3	−0.2 (2)	C9—C10—C11—C12	−1.3 (3)
C14—C1—C2—C3	−178.22 (15)	C10—C11—C12—C13	0.8 (3)
C2—C1—C6—C5	2.0 (2)	C11—C12—C13—C8	0.5 (2)
C2—C1—C6—C7	−176.27 (15)	C11—C12—C13—C14	179.27 (15)
C14—C1—C6—C5	−179.86 (15)	C8—C13—C14—C1	6.3 (2)
C14—C1—C6—C7	1.8 (2)	C8—C13—C14—C15	−173.06 (13)
C2—C1—C14—C13	171.60 (14)	C12—C13—C14—C1	−172.43 (14)
C2—C1—C14—C15	−9.1 (2)	C12—C13—C14—C15	8.3 (2)
C6—C1—C14—C13	−6.3 (2)	C1—C14—C15—N1	−4.8 (2)
C6—C1—C14—C15	172.94 (14)	C13—C14—C15—N1	174.48 (14)
C1—C2—C3—C4	−1.5 (3)	O1—C16—C17—C18	−0.49 (19)
C2—C3—C4—C5	1.3 (3)	O1—C16—C17—C20	−179.47 (17)
C3—C4—C5—C6	0.6 (3)	N1—C16—C17—C18	179.11 (17)
C4—C5—C6—C1	−2.3 (3)	N1—C16—C17—C20	0.1 (3)
C4—C5—C6—C7	176.03 (17)	C16—C17—C18—N2	0.4 (2)
C1—C6—C7—C8	3.0 (2)	C16—C17—C18—C19	−179.36 (18)
C5—C6—C7—C8	−175.34 (16)	C20—C17—C18—N2	179.32 (18)
C6—C7—C8—C9	175.57 (15)	C20—C17—C18—C19	−0.4 (3)

### Hydrogen-bond geometry (Å, °)

**Table:**

*D*—H···*A*	*D*—H	H···*A*	*D*···*A*	*D*—H···*A*
C2—H2···N1	0.93	2.20	2.840 (2)	125
C15—H15···O1	0.93	2.38	2.7463 (18)	103
